# Endoscopic membranectomy for a 2-day-old neonate with duodenal membranous atresia: the youngest case reported

**DOI:** 10.1055/a-2840-7740

**Published:** 2026-04-15

**Authors:** Dandan Guo, Lijuan Wang, Lijun Cao, Hong Xiong, Wenjuan Huang, Xiaoling Fang, Xueqiang He

**Affiliations:** 1 610828 Department of Gastroenterology, The 924th Hospital of the Peopleʼs Liberation Army Joint Logistics Support Force, Guilin, China; 2 610828 Department of Neonatology, The 924th Hospital of the Peopleʼs Liberation Army Joint Logistics Support Force, Guilin, China

**Fig. 1:**
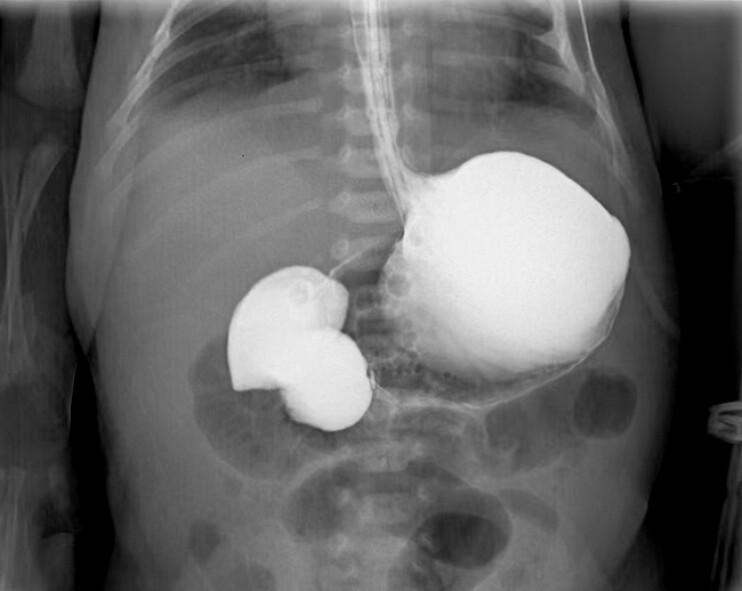
Upper gastrointestinal radiography confirmed the complete obstruction of the duodenum.

In a female infant with antenatally identified digestive tract malformations, postnatal upper gastrointestinal radiography demonstrated complete duodenal obstruction ( Fig. 1 ).

**Fig. 2:**
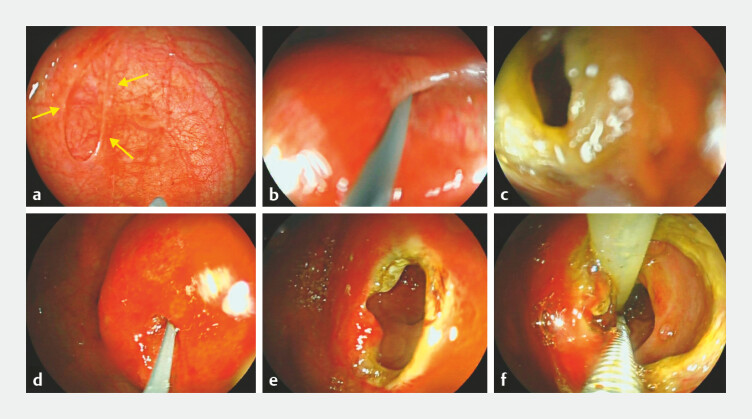
Endoscopic images. **a** Complete obstruction at the duodenal bulb‑descending junction with an oval‑shaped mucosal defect. **b** The guide wire exploration was blocked. **c** Small holes 2–3 mm in diameter were made in the mucosa. **d** Guidewire exploration identified the enteric cavity behind the mucosa. **e** A stepwise resection of the mucosa using an electrosnare eventually resulted in an opening approximately 0.8 cm in diameter. **f** The nasojejunal tube was successfully placed.

After informed consent, we performed endoscopic membrane resection on this 2-day-old infant who had no chromosomal abnormalities but presented with an atrial septal defect ( Video 1 ). Gastroscopy using a super-thin endoscope confirmed a diagnosis of duodenal membranous atresia ( Fig. 2 **a** ).

Endoscopic resection of duodenal membranous atresia.Video 1

However, the duodenal atresia site cannot be traversed by the guidewire ( Fig. 2 **b** ). Therefore, the tip of the electric snare was used to make a small incision, which was 2–3 mm ( Fig. 2 **c** ). We used a guidewire to probe and confirm the enteric cavity ( Fig. 2 **d** ). An electrosurgical snare was used to make a cruciate incision, the membrane was excised and the opening was enlarged to a diameter of 1.5 cm ( Fig. 2 **e** ). Then, diluted norepinephrine was sprayed to achieve hemostasis. Finally, we advanced a nasojejunal tube through the opening ( Fig. 2 **f** ).

**Fig. 3:**
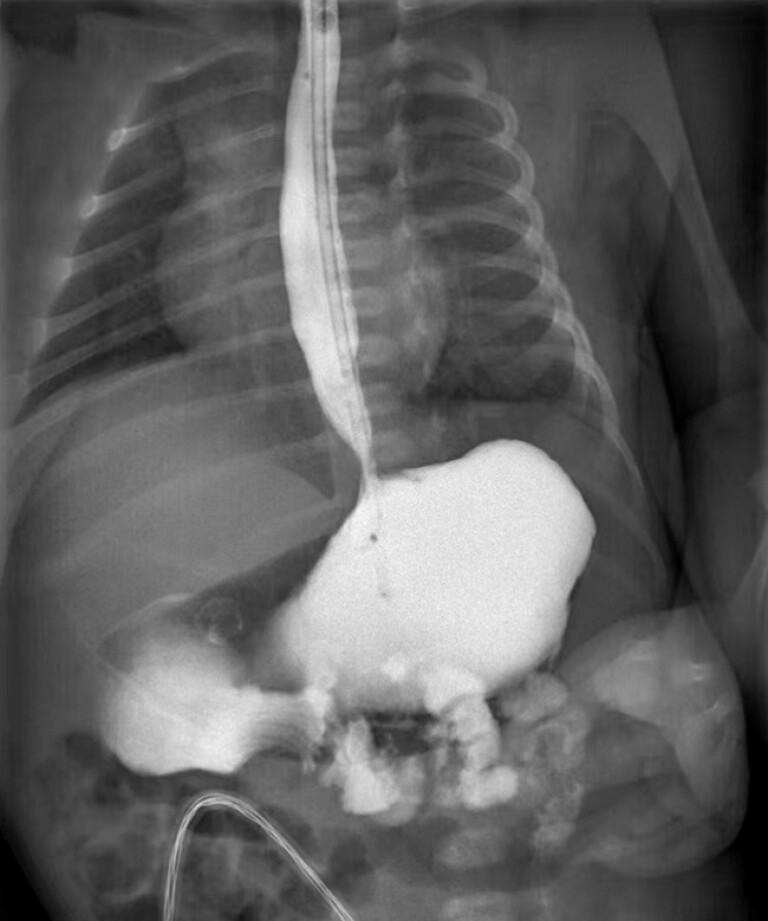
Upper gastrointestinal radiography showed the resolution of the obstruction.

On the 5th day following endoscopic treatment, upper gastrointestinal radiography revealed resolution of the obstruction ( Fig. 3 ).

**Fig. 4:**
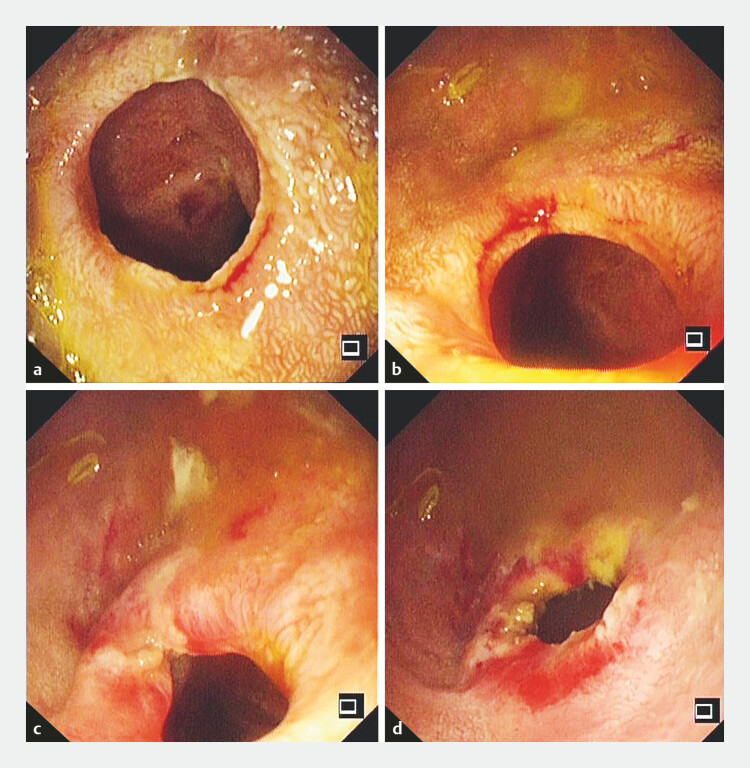
**a, b** Endoscopy showed that the original incision recovered well, and the mucosa was slightly edematous. **c, d** A small cross incision was made along the edge of the original opening, and the diameter of the mucosal opening was enlarged to 1.2 cm.

At the 20-day follow-up examination, gastroscopy revealed a patent duodenum with satisfactory mucosal recovery ( Fig. 4 **a, b** ). Multiple small incisions were made in a cross-shaped pattern at the original duodenal mucosal incision site ( Fig. 4 **c** ). The opening diameter was larger than before, expanding to 1.2 cm ( Fig. 4 **d** ). At the 3-month follow-up after endoscopic treatment, the infant was developing normally and had gained weight to 6.8 kg.

Congenital duodenal membranous atresia is rare and has traditionally been managed surgically 1 . Most reported endoscopic treatments involve older infants and children 2 3 , with neonates comprising only about 10%. The youngest patient previously reported was 7 days old 4 . We herein report the successful endoscopic management in a 2-day-old neonate, thereby extending the application boundaries of this technique.

Endoscopy_UCTN_Code_CCL_1AB_2AZ_3AZ
